# Mitochondria, mitophagy, and metabolic disease: towards assembling the puzzle

**DOI:** 10.15698/cst2020.06.222

**Published:** 2020-05-14

**Authors:** Zhiyong Chen, Marine Berquez, Alessandro Luciani

**Affiliations:** 1 Institute of Physiology, Mechanisms of Inherited Kidney Disorders Group, University of Zurich, 8057 Zurich, Switzerland.

**Keywords:** cell damage, inherited metabolic disorders, kidney tubule, metabolism, mitochondria, mitophagy, organelle quality control, oxidative stress

## Abstract

Dysregulation of the mitochondrial network in terminally differentiated cells contributes to a broad spectrum of disorders. Methylmalonic acidemia (MMA) is an autosomal recessive inborn error of intermediary metabolism caused by the deficiency of methylmalonyl-CoA mutase (MMUT) — a mitochondrial enzyme that mediates the degradation of certain amino acids and lipids. The loss of MMUT activity triggers an accumulation of toxic endogenous metabolites causing severe organ dysfunctions and life-threatening complications. How MMUT deficiency instigates mitochondrial distress and tissue damage remains poorly understood. Using cell and animal-based models, we recently discovered that MMUT deficiency disables the PINK1-induced translocation of PRKN/Parkin to MMA-damaged mitochondria, impeding their delivery and subsequent dismantling by macroautophagy/autophagy-lysosome degradation systems (Luciani et al. *Nat Commun*. 11(1):970). This promotes an accumulation of damaged and/or dysfunctional mitochondria that spark epithelial distress and tissue damage. Using a systems biology approach based on drug-disease network perturbation modeling, we predicted targetable pathways, whose modulation repairs mitochondrial dysfunctions in patient-derived kidney cells and ameliorates disease-relevant phenotypes in *mmut*-deficient zebrafish. These results unveil a link between primary MMUT deficiency, defective mitophagy, and cell distress, offering promising therapeutic avenues for MMA and other mitochondria-related diseases.

Mitochondria — the intracellular powerhouse that stores energy and signaling metabolites — are highly dynamic, double-membrane organelles that enact metabolism, hence sustaining physiology and organismal health. The maintenance of these functionally pleiotropic organelles is particularly relevant in terminally differentiated cells that are highly dependent on aerobic metabolism. Disruption of the mitochondrial network function and homeostasis might therefore confer a potentially devastating vulnerability to many different cell types, culminating in a broad spectrum of diseases.

Organic acidemias constitute a large group of inherited, life-threatening disorders of intermediary metabolism, mostly caused by deficiencies in enzymes that coordinate the amino acid degradation. Methylmalonic acidemia (MMA; MIM #251000) — the most common form of organic acidemias — is due to recessive, inactivating mutations in the *MMUT* gene encoding methylmalonyl-CoA mutase (MMUT) — a (vitamin B12-dependent) mitochondrial enzyme that catabolizes branched chain amino acids and certain lipids to succinyl-CoA, thereby feeding the tricarboxylic acid (TCA) cycle and energy metabolism. Complete or partial deficiency of MMUT activity (*MMUT*^0^ and *MMUT*^-^ subtypes, respectively) results in the accumulation of toxic organic acids (e.g., methylmalonic acid, propionic acid and 2-methylcitric acid), leading to abnormalities in the mitochondrial network that drive systemic organ dysfunctions affecting primarily the brain, eye, liver and kidney. However, mechanistically, how the enzyme deficiency begets mitochondrial distress and cell toxicity remains unclear.

As MMUT is robustly expressed within the mitochondria of kidney tubular cells, we first investigated the consequences of MMUT deficiency on the function and homeostasis of the mitochondrial network. To this aim, we analyzed the properties of mitochondria in kidney tubular cells derived from the urine of either healthy controls or MMA patients harboring loss-of-function mutations in *MMUT*. Compared to control cells, MMA patient-derived kidney tubular cells (hereafter referred to as MMA cells) exhibit an accumulation of damaged and fragmented mitochondria that aberrantly generate a large amount of reactive oxygen species (ROS) and epithelial cell distress. These dysfunctions are associated with an exaggerated production of LCN2 (lipocalin 2) — a small iron-transporting protein largely produced by kidney tubular cells following cellular damage. Such mitochondrial phenotypes are also confirmed in mouse kidney tubule cells following conditional inactivation of *Mmut* or in the kidney of mice carrying a mutant *Mmut* allele (p.Met698Lys, corresponding to the patient mutation p.Met700Lys) and a knock-out *Mmut* allele (hereafter referred to as *Mmut*^KI/KO^ mice), demonstrating the key role of MMUT in preserving the mitochondrial network homeostasis and function. However, despite the metabolic and mitochondrial abnormalities, the *Mmut*^KI/KO^ mice develop no structural changes nor interstitial inflammation nor significant kidney failure nor changes in LCN2 levels in kidneys as well as in plasma and/or urine — even in aged mutant mice — thus failing to recapitulate the kidney disease associated with MMA.

To overcome this hurdle, we established the first *mmut*-knockout zebrafish model using CRISPR/Cas9 genome editing. The zebrafish model stands out for a highly conserved integrative physiology, with recognizable organ systems performing the same physiological functions as their mammalian counterparts. For instance, the patterning of the kidney tubule is remarkably conserved in the zebrafish pronephros, including junctional complexes, a high density of mitochondria and well-developed endolysosomal system, and endocytic receptors and transporters. Furthermore, zebrafish possess orthologues of human disease-associated genes, including disorders of metabolism and physiology, while retaining the opportunities to perform (high-throughput) phenotypic screens in an *in vivo* context.

Analogous to metabolic and mitochondrial alterations reported in patient-derived and *Mmut*-deleted kidney cells, we noted that both MMA-accumulating liver and kidney of *mmut*-deficient zebrafish display altered mitochondrial morphology characterized by increased mitochondrial circularity with perturbed cristae organization. Seahorse metabolic flux analyses reveal impaired mitochondrial bioenergetics in *mmut*–deficient zebrafish when compared to control larvae. These changes are paralleled by a major mitochondrial oxidative stress, as testified by *in vivo* imaging and ratiometric confocal microscopy-based analyses of glutathione redox fluorescent signals in liver Grx1-roGFP2-labeled mitochondria, demonstrating the evolutionary conservation of this connection. Mutant zebrafish faithfully recapitulate MMA characteristic disease-relevant phenotypes, such as liver mitochondriopathy, behavioural abnormalities, and an excess of mortality. These latter phenotypes are rescued by feeding the mutant zebrafish with a low protein diet — a current supportive care strategy used in MMA patients as it prevents the accumulation of methylmalonic acid and possibly other toxic endogenous metabolites. Likewise, morphologically abnormal mitochondria were also described in kidney tubules and explanted livers of patients with MMA, strongly suggesting a link between mitochondrial dysfunctions and quality control/surveillance systems in the pathogenesis of disease.

Mammalian cells have evolved quality control and surveillance strategies to maintain the mitochondrial network homeostasis and function. These salvaging pathways rely on the execution of an evolutionary conserved, dynamic, and self-regulating mechanism called macroautophagy/autophagy that culminates in the endolysosome–mediated degradation of potentially dangerous cytosolic entities (e.g., misfolded proteins and worn out organelles, and invading pathogens). Mitochondrial autophagy/mitophagy, wherein damaged and/or dysfunctional mitochondria are sequestered within autophagosomes and delivered to endolysosomes for degradation, is selectively instigated by the recruitment and activation of the mitochondrial serine/threonine protein kinase PINK1 and E3-ubiquitin-protein ligase Parkin/PRKN to the outer mitochondrial membrane. Prolonged and/or unrepairable damage to the mitochondrial network might thus trigger the PINK1-PRKN pathway, driving multiple signalling events that culminate in the engulfment and hence degradation of damaged mitochondria by endolysosomes. Given the accumulation of MMA-affected mitochondria and higher numbers of autophagic vesicles, we hypothesized that the deficiency of the enzyme MMUT might sabotage the PINK1-PRKN-mediated priming of MMA diseased mitochondria and hence their delivery to autophagy–lysosome degradation pathways. Using the translocation of PRKN to mitochondria as a *bona fide* reporter of PINK1-PRKN-dependent priming mechanisms, we observed that MMA cells display a decrease in numbers of PRKN^+^ clusters and translocation of PRKN to damaged mitochondria, in both normal and stress-induced conditions. As a direct consequence of defective PINK1-PRKN priming mechanisms, mutant cells fail to deliver their damaged mitochondria to autophagy-lysosome degradation systems, as evidenced by the sensitive mt-Keima imaging-based assay and electron microscopy. This triggers an accumulation of MMA-diseased mitochondria, as indicated by a marked increase in overall mitochondrial proteins and ratio between mt-DNA and n-DNA under both normal and mitophagy-induced conditions. Expression of the wild-type PINK1 in MMA cells activates mitophagy-mediated degradation of diseased mitochondria, thereby averting mitochondria-derived epithelial distress and cell damage. Conversely, in human haploid HAP-1 cells, CRISPR/Cas9-mediated knockout of *PINK1* or *PRKN2* provokes mitochondrial alterations that are similar to, albeit milder than, those encountered in patient derived- and in *Mmut*-deleted kidney cells. These findings suggest that anomalies in PINK1/Parkin-mediated quality control and surveillance systems might thus intersect the mitochondrial alterations induced by MMUT deficiency to reach a high level of mitochondrial dysfunction that ultimately contributes to the pathogenesis in methylmalonic acidemia patients (**Figure 1**).

**Figure 1:**
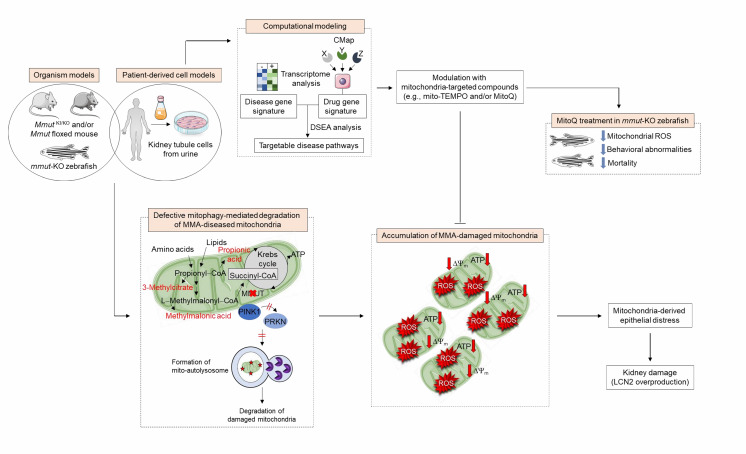
FIGURE 1: Pathophysiology and identification of druggable targets in methylmalonic acidemia. In MMA-affected kidney cells and zebrafish, deficiency of the enzyme MMUT and the resulting accumulation of toxic organic acids trigger mitochondrial alterations that are characterized by a collapse of the mitochondrial membrane potential (Δψ_m_), abnormal bioenergetics profiling, and increased generation of mitochondrial ROS and oxidative stress. Faulty execution of PINK1-PRKN-mediated mitophagy induced by MMUT deficiency impedes the delivery of damaged mitochondria and their dismantling by autophagy-lysosome degradation systems. This leads to the accumulation of dysfunctional, ROS-overproducing mitochondria that ultimately trigger epithelial distress (as evidenced by LCN2 overproduction) in patient-derived cells and disease-relevant phenotypes (as testified by liver/kidney mitochondriopathy, behavioral abnormalities and an excess of mortality) in *mmut*-deficient zebrafish. Unbiased drug–disease network perturbation modelling, based on transcriptome-wide profiles from MMA patient-derived kidney cells against a large compendium of gene signatures derived from Connectivity Map (CMAP) library of 1309 small bioactive drug compounds 1309 small bioactive drug compounds, identifies targetable disease-relevant biological pathways. Modulation of these identified targets (*e.g.*, treatment with mitochondria-targeted ROS scavengers mito-TEMPO or MitoQ) repairs mitochondrial dysfunctions, neutralizes epithelial stress and cell damage in MMA cells, and improves disease–relevant phenotypes in *mmut*–deficient zebrafish.

Isolated methylmalonic acidemias make up one of the most frequent groups of inborn errors of metabolism, which often manifest in early childhood and are associated with high morbidity and mortality. There are no curative treatments for MMA and the available therapeutic approaches can substantially decrease mortality and the overall morbidity but in most cases cannot completely prevent long-term complications. Therefore, there is an urgent need to yield promising targetable interventions in the early course of MMA.

Introducing a drug-disease network-based computational modeling approach (Mantra 2.0; Mode of Action by Network Analysis; http://mantra.tigem.it; **Figure 1**), we identified druggable pathways that might potentially reverse cellular dysfunctions associated with MMA. This cheminformatics modeling elucidates genome-wide targetable candidates by systematically matching disease gene signature, here derived from the comparison of expression profiles between MMA and their related control cells, against the Connectivity Map (CMAP) library of 1309 small bioactive drug compounds that are available for repurposing. Predictions for drug-disease pairs were based on the hypothesis that, if a drug reverses the disease gene signature, that drug might potentially target disease-relevant biological pathways. These *in silico* analyses indicate that targeting mitochondrial function and redox homeostasis might reverse disease phenotypes associated with MMUT deficiency. Inspired by the biological evidence and MANTRA predictions, we tested whether mitochondria-targeted interventions (e.g., mito-TEMPO and/or MitoQ), which rescue mitochondria-derived cell dysfunctions associated with the lysosome storage disease cystinosis, might correct disease-relevant phenotypes induced by MMUT deficiency. Treatment of MMA cells with mito-TEMPO partially restores mitochondrial network homeostasis and improves its function, normalizes mitochondrial ROS and autophagy markers, and prevents epithelial distress and cell damage. Furthermore, treatment with low doses of MitoQ alleviates the mitochondrial oxidative stress, ameliorates behavioral phenotypes and blunts the excessive mortality in the *mmut*-deficient zebrafish model of MMA. Importantly, both pharmacological interventions did not modify the levels of MMA metabolite in either MMA cells or *mmut*-deficient zebrafish, supporting the concept that mitochondrial targeting acts independently of the elevation of toxic MMA metabolites (1).

In conclusion, we identified a pathway that links a primary deficiency of a mitochondrial enzyme with dysfunction of mitophagy, accumulation of diseased mitochondria, and epithelial distress and tissue damage. These findings reveal a heretofore undescribed role of the enzyme MMUT — beyond its function in cellular metabolism — in preserving mitochondrial quality control systems and hence the integrity of specialized epithelial cells. Antioxidant compounds specifically targeting mitochondria offer novel therapeutic strategies for repairing mitochondria in MMA and other mitochondria-related disorders.

